# Muscle MRI as a biomarker of disease activity and progression in myotonic dystrophy type 1: a longitudinal study

**DOI:** 10.1007/s00415-024-12544-5

**Published:** 2024-07-07

**Authors:** Laura Fionda, Luca Leonardi, Laura Tufano, Antonio Lauletta, Stefania Morino, Gioia Merlonghi, Rocco Costanzo, Elena Rossini, Francesca Forcina, Demetrio Marando, David Sarzi Amadè, Elisabetta Bucci, Marco Salvetti, Giovanni Antonini, Matteo Garibaldi

**Affiliations:** 1 Neuromuscular and Rare Disease Centre, Neurology Unit, Sant’Andrea Hospital, Rome, Italy; 2 Department of Neuroscience, Mental Health and Sensory Organs (NESMOS), SAPIENZA University of Rome, Rome, Italy

**Keywords:** Myotonic dystrophy type 1, Muscle MRI, Longitudinal study, Biomarkers

## Abstract

**Introduction:**

Myotonic dystrophy type 1 (DM1) is an autosomal dominant disease characterized by myotonia and progressive muscular weakness and atrophy. The aim of this study was to investigate the usefulness of longitudinal muscle MRI in detecting disease activity and progression in DM1, and to better characterize muscle edema, fat replacement and atrophy overtime.

**Materials and methods:**

This is a prospective, observational, longitudinal study including 25 DM1 patients that performed at least two muscle MRIs. Demographic and genetic characteristics were recorded. Muscular Impairment Rating Scale (MIRS) and MRC score were performed within 3 months from MRIs at baseline (BL) and at follow-up (FU). We analysed 32 muscles of lower body (LB) and 17 muscles of upper body (UB) by T1 and STIR sequences. T1-, STIR- and atrophy scores and their variations were evaluated. Correlations between MRIs’ scores and demographic, clinical and genetic characteristics were analysed.

**Results:**

Eighty (80%) of patients showed fat replacement progression at FU. The median T1 score progression (ΔT1-score) was 1.3% per year in LB and 0.5% per year in UB. The rate of fat replacement progression was not homogenous, stratifying patients from non-progressors to fast progressors (> 3% ΔT1-score per year). Half of the STIR-positive muscles at BL showed T1-score progression at FU. Two patients with normal MRI at baseline only showed STIR-positive muscle at FU, marking the disease activity onset. STIR positivity at baseline correlated with fat replacement progression (ΔT1-score; *p* < 0.0001) and clinical worsening at FU (ΔMRC-score; *p* < 0.0001). Sixty-five (65%) of patients showed STIR- and fat replacement-independent muscle atrophy progression, more evident in UB.

**Conclusions:**

Muscle MRI represents a sensitive biomarker of disease activity, severity, and progression in DM1. STIR alterations precede fat replacement and identify patients with a higher risk of disease progression, while T1-sequences reveal atrophy and fat replacement progression before clinical worsening.

## Introduction

Myotonic dystrophy type 1 (DM1) is the most common muscular dystrophies in adults caused by a CTG-trinucleotide expansion in the 3´ untranslated region (UTR) of the *dystrophia myotonica protein kinase* (*DMPK*) gene [1].

DM1 is progressive multisystemic disease with skeletal and cardiac muscles primarily affected, causing myotonia and progressive muscular weakness and atrophy [2, 3].

Muscle involvement in DM1 can be easily detected by muscle magnetic resonance imaging (MRI), which represents the gold standard technique for imaging study in muscle diseases [4, 5]. Its usefulness in recognizing the pattern and severity of fat replacement, muscle atrophy and extracellular edema by T1- and T2-Short-tau-inversion-recovery (STIR)-sequences is well known, as well as its association with disease activity and progression in several muscle diseases [6–8]. We recently reported cross-sectional data from 134 DM1 patients showing that (1) fat replacement, detected by T1-sequences, correlates with clinical impairment and subclinical muscle involvement can also be detected in the milder spectrum of disease; (2) positive STIR-signal, detected in about 80% of patients, represents a marker of disease activity and seems to precede fat replacement; (3) muscle atrophy and “marbled” appearance, suggesting a premature senescence of muscle tissue, represent additional mechanisms for muscle wasting and weakness [9]. Only few studies investigated quantitative muscle MRI (qMRI) in DM1 and only one study reported longitudinal MRI data for short periods of FU observation [10, 11].

In the gene-therapy era [12], objective evaluation of natural history of progressive diseases and their correlations with clinical evolution overtime are warranted. Longitudinal studies of muscle MRI represent a potential biomarker in monitoring the natural history of disease progression and better understand the pathophysiological mechanism of disease in DM1, but it could also represent a sensitive outcome measure of response to treatment for forthcoming clinical trials, as it occurs in other myopathies [13–15].

In this framework, we aimed to investigate the value of longitudinal muscle MRI study as a biomarker and prognostic factor of disease activity and progression in DM1.

## Materials and methods

### Patients

This is a prospective, observational, longitudinal study including DM1 patients belonging to Sant’Andrea Hospital of Sapienza University of Rome. It was carried out with previous patients’ informed consent and in compliance with ethical standards of local ethical committees, the Helsinki Declaration, and the Good Clinical Practice.

All patients had genetically confirmed DM1 and performed two MRI studies including STIR and T1 sequences between July 1st, 2016, and January 1st, 2023, for routine diagnostic and clinical purposes.

Demographic and clinical data were collected for each patient.

Molecular diagnosis of DM1 was performed on DNA obtained from peripheral leukocytes at the moment of clinical diagnosis. Patients were grouped into three expansion classes according to CTG expansion range: E1 (CTG 50–150), E2 (CTG 151–1000) and E3 (CTG > 1000).

Patients were also stratified into five clinical forms based on first disease-related symptoms onset: (I) congenital (at birth), (II) infantile (1–10 years), (III) juvenile (11–20 years), (IV) adult (21–40 years) and (V) late onset (> 40 years).

All patients received a complete neurological examination, including an extensive manual muscle test scored by the Medical Research Council (MRC) scale evaluating arm abductors, forearm flexors and extensors, finger extensors, hip flexors, tight flexors and extensors, foot flexors and extensors bilaterally (MRC-100), as part of their clinical follow‐up, and Muscular Impairment Rating Scale (MIRS) [16] was calculated at each visit scheduled for clinical purposes. For this study, we programmed a clinical evaluation within 3 months from each muscle MRI assessment.

### Muscle MRI studies

All muscle MRIs were obtained using a 1.5-T MRI following previously described protocols [17, 18] in accordance with the international consensus recommendations [19]. A total of 32 muscles of lower body (LB) and 17 muscles of upper body (UB) from each side were analysed. LB included lower limbs, pelvic girdle, abdominal muscles and lumbar paraspinal muscles whereas UB included head, neck, scapular girdle and chest muscles. T1 Turbo spin echo (T1-TSE) and T2-Short tau inversion recovery (T2-STIR) sequences were obtained in each study with axial, coronal and sagittal planes. The following muscles were grouped and evaluated together because of the difficulty in evaluating them independently in most cases: *obliquus abdominis externus/internus‐transversus abdominis; obturatorius externus/internus; extensor digitorum/hallucis longus; peroneus longus/brevis; tibialis posterior/flexor digitorum longus; pterygoideus internus/externus; cervical paraspinal muscles; thoracic paraspinal muscles; teres major/minor*.

The duration of the MRI exam was approximately 50 min.

Axial T1 sequence (TR/TE of 400/13 ms, thickness/gap 4 mm/0, 4 mm, field of view [FOV] 370 mm) was performed by two contiguous stacks to obtain an anatomic coverage from the skull base to the ankles. STIR axial images (TR/TE/TI 3000/35/160 ms) were also acquired with the same anatomical coverage of T1 sequences. For the upper body, T1 sequences also included coronal (TR/TE of 450/13 ms, thickness/gap 3.5 mm/0.35 mm, FOV 400 mm) and sagittal (TR/TE of 630/13 ms, thickness/gap 5 mm/0.5 mm, FOV 400 mm) acquisitions to better evaluate the neck and thoracic muscles. Three coronal stacks were obtained to assess anterior and posterior thoracic muscles and neck muscles. Slices were oriented along (I) the axis of the pectoralis major muscle for anterior thoracic muscles, (II) parallel to the dorsal kyphosis for posterior thoracic muscles and (III) the major axis of the neck for neck muscles. Sagittal stack was oriented to cover from one shoulder to the other.

Fat replacement was assessed for each muscle, evaluated throughout the whole muscle bulk, on T1 sequences using a six-point scale (0–5) according to modified Mercuri classification [20] as previously reported, while edema/inflammation on STIR sequences using a two-point scale (0: negative, 1: positive).

The degree of muscle atrophy was evaluated by a semiquantitative five-point scale in lower body considering posterior, medial and anterior compartments of the thigh, and anterior and posterior compartments of the leg [9, 21]. In the upper body muscle atrophy was assessed for four isolated muscles: *sternocleidomastoideus*, *trapezius*, *pectoralis minor* and *major* [9].

We obtained the overall percentage of STIR positivity as a fraction of STIR-positive muscles over the total number of muscles evaluated (STIR% = total n°STIR-positive muscles/total muscles evaluated*100). STIR% was also calculated separately in UB and LB (STIR%UB and STIR%LB).

The overall burden of fat replacement was obtained as a sum of T1 score of each muscle per patient (T1-score) ranging between 0 (no fat replacement at all) and 485 (the value corresponding to the max T1-score = 5 for all muscles: 97 × 5 = 485). We also calculated the T1-score for UB and LB independently (T1-UB score and T1-LB score, respectively). We obtained the percentage of T1-score as a fraction of the sum of T1 scores of all muscles over the value of max T1-score for all muscles per patient (T1% = sum of T1-score of all muscles/sum of max T1-score in all muscles*100).

For longitudinal evaluation of MRI changes, we evaluated the variation of STIR% and T1-score over time (ΔSTIR% and ΔT1% scores respectively), between the first and last MRI available for each patient. We also analysed ΔSTIR% and ΔT1% scores for UB and LB independently.

T1 and STIR sequences of head, scapular girdle and trunk were not available for one patient at baseline and for one patient at follow-up.

Two independent Neurologists with experience in MRI analysis (MG, LF) blinded to demographic and clinical data analysed all MRIs. In muscles with different T1 and STIR scoring, observers reviewed the muscles together to agree the final score.

### Statistical analysis

Continuous variables were expressed as mean, range, and inter quartile range (IQR).

We identified that none of the variables analysed was normally distributed using Kolmogorov–Smirnov and, therefore, we used non-parametric statistical studies. Mann–Whitney *U* test was used to identify whether differences observed between two groups were significant. Wilcoxon signed rank test was used to determine whether differences observed in continuous variables at two time points (BL and FU) were statistically significant (e.g., T1-LB score at BL and at FU). When multiple comparisons were performed, we applied a post hoc Bonferroni correction. Chi-square test was used for comparison of categorical variables. Spearman rank-order test was run to assess if correlations between variables were statistically significant. Correlation coefficients are expressed as *r* and considered strong correlation if higher than 0.8 and good if higher than 0.6. Two-sided *p* values were calculated for all analyses; values of < 0.05 were considered significant. All these analyses, as well as the graphics development, were performed using JASP Statistics 0.16 (IBM, Armonk, New York, USA) and GraphPad Prism 8.2.1.

## Results

### Patients

Twenty-five patients (13 males, 12 females), aged between 18 and 81 years (mean 41, SD ± 16), were included in the study.

Median age at disease onset was 27 (IQR 16–43, range 0–81).

CTG average expansion range was 50–1250 (median 475, IQR 308–575). Four patients were in E1 expansion class, 19 in E2 and 2 patients in E3.

Median disease duration at first MRI study was 9 years (IQR 3.5–16.5, range 0–43), with three patients still asymptomatic, while at the second MRI was 11 years (IQR 6.5–20, range 2–45). Median follow-up duration was 37 months (IQR 32–47, range 21–53) between the first and the second MRI.

MIRS evaluation at BL showed a mean value of 2.84 (SD ± 0.75), while at FU it was 3.04 (SD ± 0.89). Median MIRS was 3 at baseline (range 1–4, IQR 2–3) and at follow-up (range 1–5, IQR 2.5–4). At first MRI, 1 patient had MIRS 1, 6 patients had MIRS 2, 14 patients had MIRS 3, 4 patients had MIRS 4. At follow-up, MIRS progression was observed in 5 patients (one MIRS 2 to 3; three MIRS 3 to 4; one MIRS 4 to 5).

Overall median MRC at BL was 93,3 (range 76.5–100, IQR) while at FU it was 92 (range 37.5–100).

Major clinical and MRI characteristics of each patient are summarized in Table 1.

**Table 1:** Major clinical and MRI characteristics of each DM1 patient

	sex	Age at disease onset (years)	CTG expansion class	CTG average expansion	Disease duration (years)	Age at 1st MRI (years)	Age at 2nd MRI (years)	Follow-up duration (month)	MIRS at 1st MRI	MIRS at 2nd MRI
*P1*	M	0	2	825	43	43	45	32	4	4
*P2*	F	20	2	475	5	25	27	21	2	2
*P3*	M	51	1	70	4	55	59	53	2	2
*P4*	F	16	2	445	11	27	31	42	3	3
*P5*	F	19	2	325	10	29	33	42	3	3
*P6*	M	18	2	575	2	20	23	37	3	3
*P7*	M	47	2	210	10	57	60	37	4	5
*P8*	M	53	2	575	1	54	57	37	3	3
*P9*	F	0	3	1250	26	26	31	50	3	4
*P10*	F	0	3	1000	18	18	22	50	3	3
*P11*	M	16	2	485	15	31	34	37	3	4
*P12*	M	30	2	500	24	54	57	42	4	4
*P13*	M	35	2	425	6	41	43	33	2	3
*P14*	F	19	2	750	34	53	57	50	4	4
*P15*	M	28	2	560	8	36	39	38	3	4
*P16*	F	42	1	73	0	42	44	30	1	1
*P17*	F	29	2	292	11	40	44	48	3	3
*P18*	M	75	1	62	0	75	79	49	2	2
*P19*	F	81	1	50	0	81	84	32	2	2
*P20*	M	12	2	500	26	38	40	25	3	3
*P21*	M	44	2	350	3	47	50	35	3	3
*P22*	F	35	2	350	8	43	46	35	3	3
*P23*	F	27	2	425	6	33	35	25	3	3
*P24*	F	24	2	640	9	33	35	25	3	3
*P25*	M	10	2	489	13	23	27	46	2	2

### STIR and T1 analyses

At BL, 19 patients (76%) showed at least two STIR-positive muscles. The mean STIR% score was 12.8% in lower body (STIR%LB) (range 0–39.1%, IQR 1.6–18) while in upper body (STIR%UB) was 1.6% (range 0–9.1%, IQR 0–1.5). FU MRI analysis showed STIR alterations in at least two muscles in 22 patients (88%), with a mean STIR%LB of 20.1% (range 0–40.6%, IQR 10.2–31.7) and median STIR%UB score of 5.3% (range 0–48.5%, IQR 0–7.6), and an overall STIR% mean score of 14.9% (range 0–43.3, IQR 7.7–21.1). Global ΔSTIR% was 6% between first and last MRI (range 0–25.8%). Two patients with completely normal MRI at baseline (STIR negative, T1 negative) showed STIR positivity in some muscles of the legs at FU without fat replacement.

**Fig. 1:**
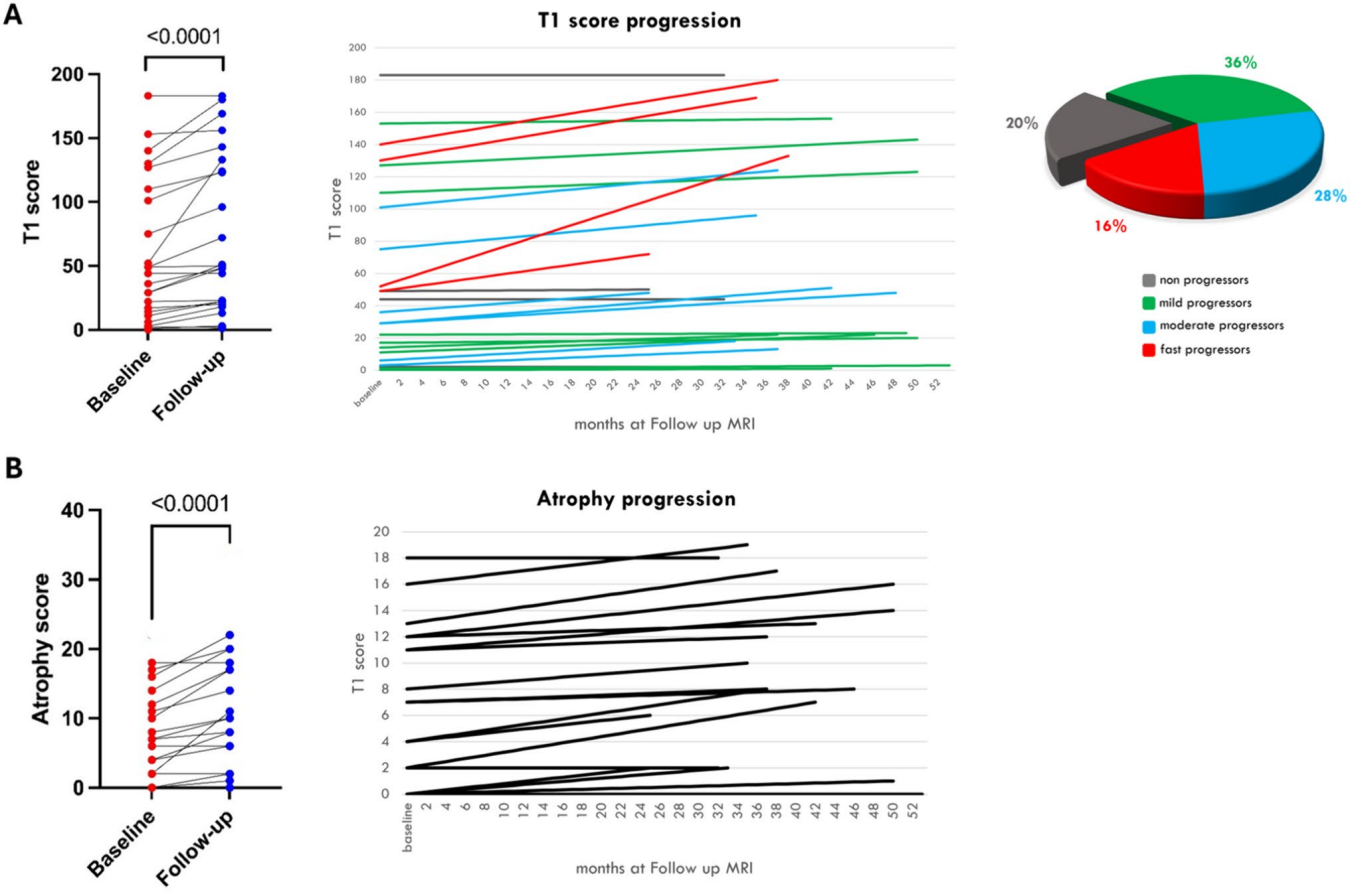
T1-score and Atrophy-score progression. On the left, overall T1- (**A**) and Atrophy-score (**B**) variation at FU for each patient. In the middle, T1- (**A**) and Atrophy-score (**B**) variation for each patient according to months at follow-up MRI. On the right, graphic representation of percentage of non-, mild-, moderate- and fast progressors

T1 sequence analysis revealed mean T1-score of 55.4 at BL (range 0–183, IQR 8.5–105.5) and a mean T1-score of 69.8 at FU (range 1–183, IQR 19–128.5), corresponding to a mean progression of overall T1-score (ΔT1%) of 3.1% (range 0–16.7%). Mean overall progression was 3.9% for LB (ΔT1-LB = 1.3% per year) (range 0–19.1%) and 1.5% for UB (ΔT1-UB = 0.5% per year) (range 0–12.1%) muscles. Considering all patients, the mean ΔT1%-LB per year was 1.28%, ranging from 0 to 6.03%. There was a significant progression of T1-score (*p* < 0.0001) and T1% (*p* < 0.0001) at FU considering all muscles. Five patients (20%) did not show any T1-score progression (ΔT1 = 0). In these patients the mean time-lapse between MRIs was significantly shorter compared to the twenty patients who progressed (28 months vs 40 months respectively, *p* = 0.003). By contrast, among the 20 patients showing some degree of fat replacement progression, the % of T1-score progression per year differs among patients. Nine patients (36%) showed a mild progression (0–1% T1-score progression per year), seven patients (28%) showed a moderate progression (1–3% per year) and four patients (16%) showed a fast progression (> 3% per year) (Fig. 1A). The rate of fat replacement progression did not show any correlation with disease severity and duration, age, gender or genetical background.

The leg muscles showed the higher T1-score progression at FU. Muscles with higher degree of progression of fat replacement (> 3%) in LB were: *gastrocnemius medialis* (mean ΔT1-score% + 10–11%), followed by *soleus* (7–10%), *flexor hallucis longus* (8%), *gastrocnemius lateralis* (7–8%), *tibialis anterior* (6–8%), *vastus medialis* (6–8%) and *iliocostalis lumborum* (4–6%), followed by the lumbar paraspinal muscles, *biceps femori caput breve*, *rectus abdominis, semitendinosus, tensor fasciae latae, sartorius* and *gluteus minimus*. In UB *trapezius* (4–6%), *genioglossus* (4%) and cervical paraspinal muscles (3–4%) showed the higher degree of percentage of T1 fat replacement progression.

### STIR-dependent T1-score progression

**Fig. 2:**
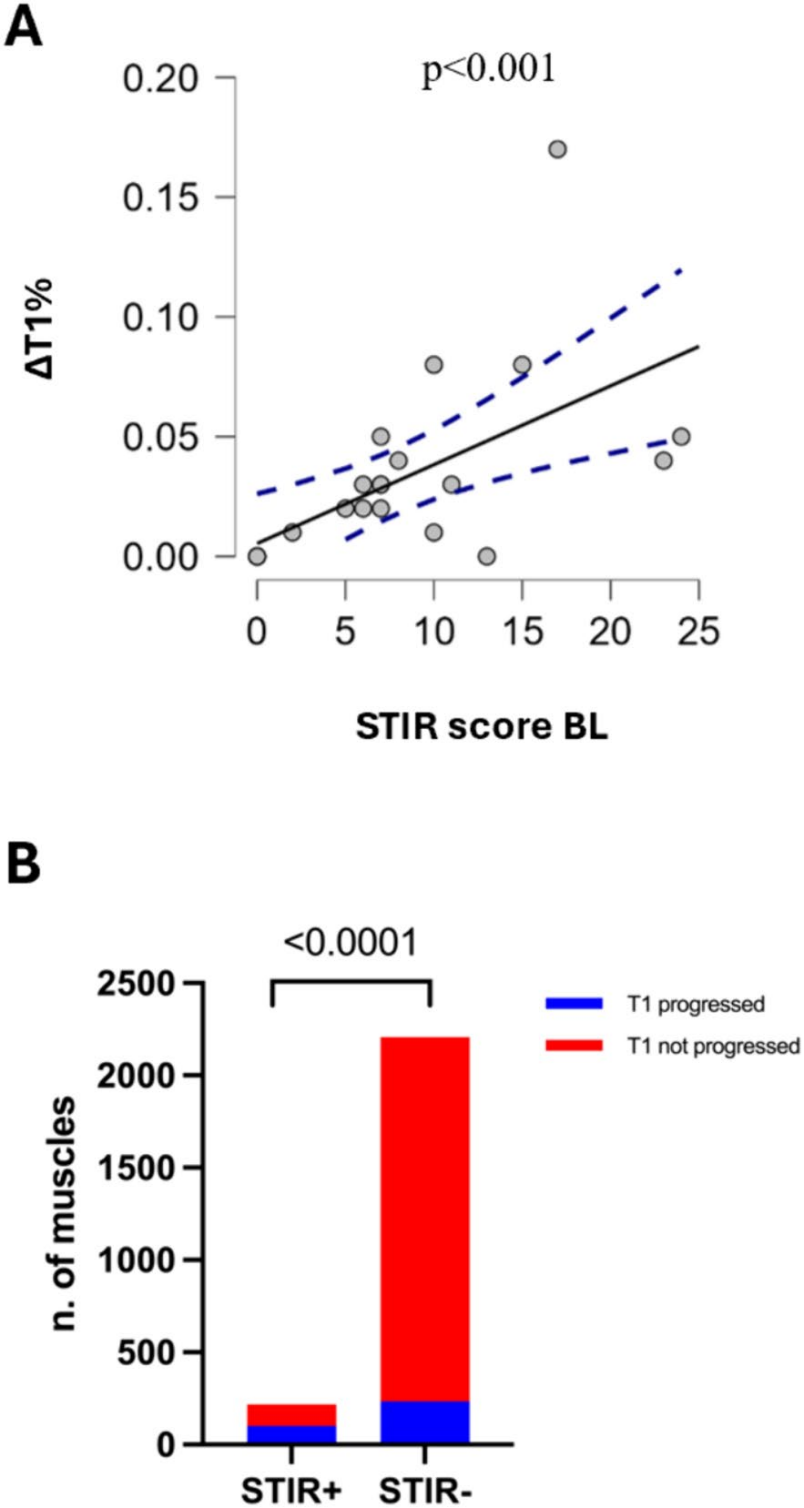
STIR-dependent fat replacement progression. **A** significant correlation between STIR positivity at BL and fat replacement progression at FU (ΔT1%). **B** Number of muscles progressed (blue) and not progressed (red) in T1 sequences at FU, accordingly to STIR positivity/negativity at 1st MRI

T1-score progression significantly correlated with STIR% at BL (*p* < 0.001, *r* = 0.76) regardless of disease duration (Fig. 2A). The average ΔT1%-LB per year significantly correlated with STIR- LB score at BL (*p* < 0.001, *r* = 0.69). In particular fast progression (ΔT1-LB score per year > 1%) and ultra-fast progression (ΔT1-LB score per year > 3%) significantly correlated with STIR-score at BL regardless of FU duration (*p* < 0.001, *r* = 0.86 and *p* < 0.001, *r* = 0.63, respectively). Conversely, no significant correlation was found between non-progression (ΔT1-score LB = 0%) and any clinical or MRI features.

**Fig. 3:**
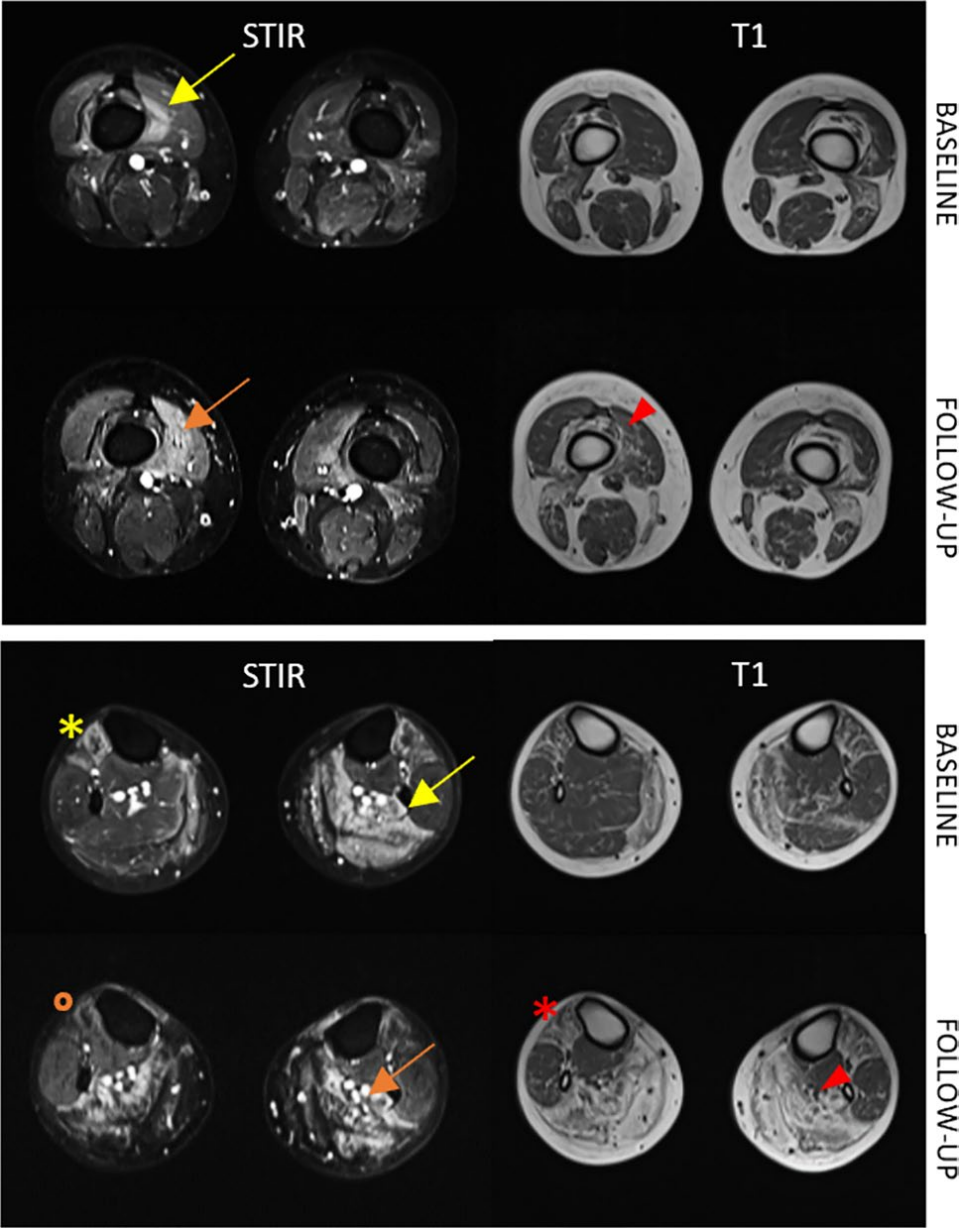
Muscle MRI findings. Axial plane of lower limbs in STIR and T1 sequences at BL and FU: STIR-positive right vastus medialis and left soleus at BL (yellow arrows) showed fat replacement progression in T1 sequences at FU study (red arrowheads) and still STIR-positive signal at FU (orange arrows); similarly STIR-positive right tibialis anterior at BL (yellow asterisks) showed T1 progression with complete fat replacement (red asterisks) but STIR negative (orange dot) at FU (P15, 36 years-old male at first MRI, FU duration 38 months, MIRS 3, E2 expansion class)

Overall, 218 muscle were STIR positive at BL, 102 of which (46.8%) showed some degree of T1-score progression (at least 1 point of Mercuri scale) at the 2nd MRI study. Of the 2207 STIR-negative muscles at BL, 235 (10.7%) showed a T1-score progression at FU (*p* < 0.0001) (Figs. 2B and 3). On the other hand, the total amount of muscles that showed T1 progression at FU, regardless of STIR positivity at first MRI, was 337 muscles. Of these, 102 were STIR positive at the BL (30.2%) while 235 were STIR negative. Interestingly, among the 235 STIR-negative muscles at BL that showed T1 progression, 71 became STIR positive at FU.

**Fig. 4:**
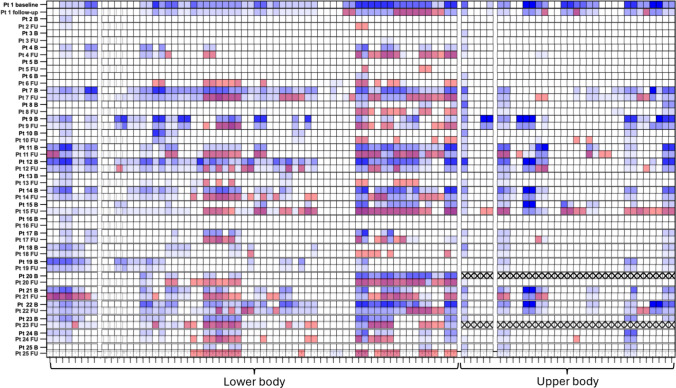
Segmental Heatmap. Overlapped STIR positivity (red or not) and T1-score (blue scale) of at BL and at FU for each patient and each muscle; note the good overlapping between T1 progression (darker blu) at FU and STIR positivity at BL and FU

The heatmap analysis shows a good overlap between STIR-positive muscles and fat replaced muscles in T1 (Fig. 4).

### STIR-independent atrophy progression

Sixteen patients showed atrophy in at least one muscle or compartment at BL, 19 at follow-up. Mean value of overall atrophy was 6.4 at BL (range 0–22) and 9.2 at FU (range 0–33) (*p* < 0.0001).

At BL, 17 patients showed some degree of atrophy of at least one UB muscle, while only 7 patients had atrophy of at least one muscle of LB compartments. At FU, 18 patients showed atrophy in UB, while 13 patients had some degree of atrophy of at least one LB compartment.

All patients showing muscle atrophy progression had some degree of muscle atrophy in at least one UB muscle at BL. Considering LB, only seven patients showed atrophy in at least one compartment at BL.

Overall mean progression was 4% (range 0–15.2%) (*p* < 0.0001), 5% in UB (range 0–35%) while in 3% in LB (range 0–20%) (Fig. 1B).

**Fig. 5:**
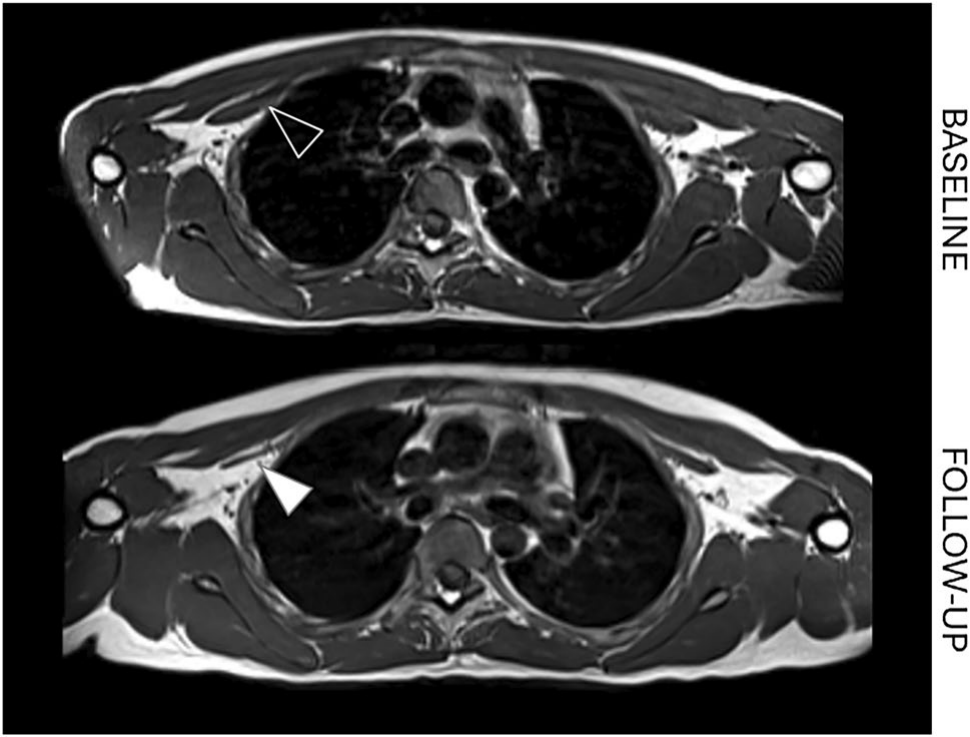
Atrophy progression. STIR-independent atrophy-progression of pectoralis minor at first (up) and last (down) MRI study after 37 months (P6, 20 years-old male, FU duration 37, months, MIRS 3, E2 expansion class)

Fifteen out of twenty-three patients (65%) showed some degree of atrophy progression in UB at FU. None of the muscles of UB that showed an atrophy progression at FU was STIR positive at BL (Fig. 5). Conversely, in LB, atrophy progression was also associated with fat replacement progression. Thigh atrophy was detected in right vastus medialis of two patients at baseline, both showed a 1-point progression during FU. One showed 1-point T1 progression (2 to 3) and also STIR positivity at BL and FU; the other showed no fat replacement at both MRIs, neither STIR hyperintensity. One more patient developed thigh atrophy at FU.

Atrophy-score in UB at BL and FU significantly correlated with MIRS score (*p* < 0.001).

### Clinical-MRI correlations

Overall T1-score at BL correlated with disease duration at both MRI studies (*p* = 0.007, *r* = 0.52) and also with MIRS at BL and at FU (*p* < 0.0001, *r* = 0.72 and 0.75).

T1 score at FU correlated with disease duration (*p* = 0.007, *r* = 0.52). Both T1-score at FU and ΔT1-score correlated with MIRS at BL and at FU (*p* < 0.0001, *r* = 0.77 and *p* = 0.009, *r* = 0.51, respectively). Noteworthy among the 80% of patients who showed some degree of T1-score progression at FU, only 16% showed MIRS progression at FU. MIRS progression at FU significantly correlated with ΔT1-score (*p* = 0.004, *r* = 0.57) and STIR-score at BL (*p* = 0.04, *r* = 0.41), but not with atrophy score.

In general, we did not find any correlation between T1 score variation (ΔT1-score) and time-lapse between MRIs.

STIR% at BL correlated with disease duration at first MRI (*p* = 0.007, *r* = 0.53). STIR% at BL showed also a significant correlation with MIRS at both BL (*p* = 0.001 *r* = 0.72) and FU (*p* < 0.0001, *r* = 0.7). Interestingly also STIR% at FU showed a good correlation with MIRS at FU (*p* = 0.001, *r* = 0.61).

MRC at BL and at FU correlated with T1-score at both timepoints (*p* < 0.0001, *r* = 0.74 and *r* = 0.75). MRC also correlated with atrophy scores, at BL (*p* < 0.0001, *r* = 0.85) and at FU (*p* < 0.0001, *r* = 0.86), and with STIR% at BL (*p* < 0.0001, *r* = 0.74) and at FU (*p* < 0.0001, *r* = 0.77).

For correlation analysis we also evaluated LB-MRC (MRC-60) with respect to T1-LB score and we found a significant correlation at BL (*p* < 0.0001, *r* = 0.74) and at FU (*p* < 0.0001, *r* = 0.78).

ΔT1-score only correlated with MRC-100 at FU (*p* < 0.05, *r* = 0.5), while ΔMRC correlated with T1-score at BL and at FU (*p* < 0.05, *r* = 0.42 and *r* = 0.45) but not with ΔT1-score.

CTG average expansion correlated with age at disease onset (*p* < 0.0001, *r* = − 0.72), with MIRS at BL (*p* = 0.01 *r* 0.5) and at FU (*p* = 0.008 *r* = 0.52), and with MRC-100 at BL (*p* < 0.001, *r* = 0.68) and at FU (*p* < 0.01, *r* = 0.62), but did not correlate with any MRI parameters.

Linear regression analysis showed that there were no correlation between ΔT1% with time-lapse between MRIs, age, age at disease onset, CTG expansion and disease duration.

## Discussion

This prospective, longitudinal study provides 3-year FU data on a cohort of clinically and genetically characterized DM1 patients, combining semiquantitative T1 muscle MRI evaluation for fat replacement and muscle atrophy, and the assessment of STIR hyperintensity, with genetic and clinical measures. Natural history of fat replacement and muscle damage in DM1 patients is of key importance for upcoming clinical trials, especially for the role that muscle MRI could have as a biomarker of disease progression. To date, there are no longitudinal MRI studies in DM1 patients.

Our results showed significant changes in all MRI parameters evaluated and a significant correlation between MRI and clinical characteristics, such as disease duration, MRC and MIRS. We found that fat replacement progression was more evident in LB (1.3% of T1-score per year) compared to UB (0.5% per year) but conversely that atrophy progression was more evident in UB (5%) compared to lower body (3%).

Interestingly we observed that the rate of fat replacement progression differs among patients, from non-progressors (ΔT1-score = 0) to fast progressors (> 3% per year). These findings does not correlate with any demographic, clinical or genetical background, suggesting other underlying mechanisms influencing disease progression in DM1. Moreover, it could be hypothesized that disease progression in DM1 could not have a constant rate along life, but vary with periods of stability and periods of progression. Nevertheless, this hypothesis have to be supported by further evidences.

Muscles with a higher rate of fat replacement progression are the earliest affected muscles, according to the pattern of muscle involvement in DM1, suggesting that some muscles (e.g. *gastrocnemius medialis*, *quadriceps*) are the most sensitive muscles in monitoring disease onset and progression, as also previously reported [9, 22], in particular *gastrocnemius medialis* for the early stages of disease (including asymptomatic and pre-symptomatic) and *quadriceps* for the advanced stages of the disease.

Another important aspect of the study is the confirmation of the sensitivity and specificity of muscle MRI in predicting fat replacement and disease progression. In fact, we found a strong correlation between fat replacement progression and MIRS progression. Moreover, we found a good correlation between STIR positivity at baseline and MIRS progression at FU. Noteworthy, we observed that only 20% of patients showing T1-score progression also had a MIRS progression at FU, suggesting that muscle MRI is more sensitive than clinical scales in detecting disease activity and progression. Moreover, since we used a semiquantitative scale for muscle MRI scoring, we were able to catch only the major MRI changes, able to modify the Mercuri score, probably underestimating the power of a quantitative imaging technique in detecting even minimal disease progression.

We observed a strong correlation between STIR positivity at baseline (STIR%) and both fat replacement progression (ΔT1) and clinical worsening (ΔMRC) suggesting the high sensitivity of STIR positivity as a prognostic indicator of high risk of disease progression.

This study confirms the hypothesis we proposed in our first cross-sectional study [9] concerning the fat replacement progression of STIR-positive muscles. We found a strong correlation between STIR positivity at BL and fat replacement progression at FU, demonstrating that STIR positivity precedes fat replacement also in DM1, as it occurs in other muscular dystrophies or inflammatory myopathies [23–25]. STIR hyperintensity also shows the same pattern of muscle involvement of T1, as shown by the heatmaps and had a good correlation with clinical scales at both timepoints.

Almost half of STIR-positive muscles at BL showed some degree of T1-score progression at FU, while only 10% of STIR-negative muscles at BL showed a T1-score progression at FU. Interestingly 30% of these muscles were STIR positive at FU, suggesting that they became STIR positive some time between the first and second MRI, supporting the evidence that STIR positivity is associated with fat replacement. For the remaining muscle that showed STIR-independent progression of fat replacement, the most probable explication is that semiquantitative T1 scoring is not enough to detect minimal changes, which could have been probably detected by quantitative Dixon acquisition. Alternatively, other hypothesis can be made: (1) STIR positivity could occur for very short periods not enough to be detected by MRI studies lasting 3 years of FU, but influencing fat replacement progression; (2) other pathophysiological mechanisms leading to fat replacement occurs in muscles bypassing pathways causing STIR positivity; (3) low MRI observer reliability.

One other important piece of evidence about STIR sequences is that among patients with normal muscle MRI at baseline (STIR negative, T1 negative), two of them showed only STIR-positive muscle at FU in the legs, identifying for these patients the onset of disease activity, marking the transition from an asymptomatic stage of disease to a pre-symptomatic stage of disease.

On the other hand, half of STIR-positive muscle did not show fat replacement progression. It could be explicated by the fact that the rate of fat replacement progression could differ among muscles, and some muscles could require longer observation periods to detect fat replacement progression; moreover, STIR positivity could start in proximity of MRI study, and time-lapse between two MRI studies could not be enough to lead to fat replacement; finally, STIR positivity could be a reversible process, interrupted by other endogenous or exogenous mechanisms before fat replacement occurrence.

What there is behind STIR positivity in DM1 is not known since there is no evidence of an inflammatory process behind muscle degeneration in DM1 muscle biopsies. Anyway, STIR positivity reflects not only inflammation, but also muscle edema, which could also be the consequence of necrosis and regeneration, or vascular or neurogenic alteration [5, 26, 27]. It represents a reversible marker of disease activity, often predicting T1 changes over time and it is associated with clinical worsening. For this reason, STIR hyperintensity could be useful in identifying those muscles potentially susceptible of targeted disease-modifying therapies, representing a sensitive biomarker of early disease activity in DM1 for upcoming clinical trials.

Finally, muscle atrophy could represent another important mechanism underlying muscle weakness in DM1. In fact, we observed that muscle atrophy progression is independent of STIR positivity and fat replacement in UB, with respect to the LB where the atrophy follows fat replacement. Both atrophy and atrophy progression are more evident in UB muscles, especially *trapezius*, *sternocleidomastoideus*, and *pectoralis minor*. This study confirms that in UB, muscle atrophy and atrophy progression are independent from fat replacement, suggesting additional mechanisms of muscle wasting in DM1. By contrast, in LB, muscle atrophy is associated with fat replacement, as it occurs in other muscular dystrophies and spinal muscular atrophy [21, 28, 29]. In LB, atrophy score degree and progression was more evident for leg posterior compartment, following the distribution of STIR hyperintensity and T1 fat replacement; anyway, also *tibialis posterior* volume was previously found to decreased overtime in DM1 patients compared to healthy subjects [22].

Even if we observed a good correlation between atrophy score in UB and clinical severity, as observed by other authors [30], no correlation with disease progression or clinical worsening were found, suggesting that more data are needed to establish whether or not it could represent a good parameter for disease progression. Of note, atrophy evaluation included *sternocleidomastoideus*, *trapezius*, *pectoralis minor* and *major* on the basis of previous findings [9]: there can be also considered trunk muscles, with an antigravity action, known to have a good correlation with postural stability or balance in patients with muscular dystrophies [31, 32]. However, differently from our findings, these studies found a continuum between fat infiltration, edema and muscle size in trunk muscles analysed. It could be explained by the difference in evaluated muscles: maybe abdominal muscles, *erector spinae* and *psoas* follow the same pathological changes of LB muscles of our study, in which T1 fat infiltration seems to have a role in atrophy progression, while *sternocleidomastoideus*, *trapezius*, *pectoralis minor* and *major* undergo atrophy without additional MRI changes, maybe following different pathological mechanisms.

A last consideration concerns the influence of disease duration on disease progression. In our cohort, even if non-progressors had a significantly shorter FU, we did not find any correlation between time-lapse between MRIs and T1 score variation (ΔT1-score), suggesting that other factors independent by disease duration influence the rate of fat replacement and disease progression in patients. This data let us suppose that in the natural history of the disease, periods of disease activity and progression could alternate with periods of disease quiescence, possibly influenced by endogenous or exogenous factors, as it occurs in disease onset, after a long period of quiescence after birth. Moreover, no correlations were found between MRI changes overtime and CTG expansion. Even if some cross-sectional studies found a correlation between MRI alterations and CTG expansion [33, 34], we evaluated fat replacement progression (i.e. ΔT1%) overtime in patients with different age, in different stages of the disease, in which the occurrence of somatic mosaicism and triplet repeat expansion instability could even increase the diversity, also considering that the genetic diagnosis and MRI studies were performed at different times.

This study has several limitations that deserve to be mentioned: (1) the first, and most important, is the use of a semiquantitative scoring of MRI: even if we have been able to demonstrate the higher sensitivity of MRI with respect to clinical evaluation and clinical scales to detect disease activity and progression, quantitative MRI acquisition would be even more sensitive to detect minimal changes before clinical worsening and should be preferred for clinical trials; (2) small sample size: clinical and MRI data could be influenced by several factors such as phenotypical variability, age of patients, disease duration and stage, that could underestimate some MRI-clinical association because of lacking statistical power; (3) inhomogeneous FU duration: even if we normalized the MRI findings for time-lapse between MRIs, clinical assessment and correspondent MRI features would have been more comparable and reliable between patients with even more similar observation period; (4) clinical and genetic heterogeneity of the sample, in terms of disease severity at baseline and CTG average expansion.

## Conclusion

Muscle MRI is a sensitive biomarker of disease activity, severity, and progression in DM1 and it is more accurate than clinical evaluation, also for the milder spectrum of disease. Fat replacement and progression in more evident in the LB, while muscle atrophy progression is more evident in UB regardless of STIR positivity and fat replacement. STIR positivity precedes fat replacement and represents a biomarker of disease activity, also in the early stages of the disease, helping to identify the disease onset in asymptomatic patients. STIR positivity at BL correlates with fat replacement progression and clinical worsening at FU, identifying patients with higher risk of disease progression. It can identify those muscles with a potentially reversible pathological process, representing a promising biomarker of disease activity. Muscle MRI assessment should be introduced routinely in forthcoming clinical trials’ design, representing a sensitive and reliable biomarker able to capture minimal muscle changes in a short period.

## Data Availability

Data related to this study are available upon reasonable request.
