# Correction: Muscle MRI as a biomarker of disease activity and progression in myotonic dystrophy type 1: a longitudinal study

**DOI:** 10.1007/s00415-024-12886-0

**Published:** 2025-02-01

**Authors:** Laura Fionda, Luca Leonardi, Laura Tufano, Antonio Lauletta, Stefania Morino, Gioia Merlonghi, Rocco Costanzo, Elena Rossini, Francesca Forcina, Demetrio Marando, David Sarzi Amadè, Elisabetta Bucci, Marco Salvetti, Giovanni Antonini, Matteo Garibaldi

**Affiliations:** 1 Neuromuscular and Rare Disease Centre, Neurology Unit, Sant’Andrea Hospital, Rome, Italy; 2 Department of Neuroscience, Mental Health and Sensory Organs (NESMOS), SAPIENZA University of Rome, Rome, Italy

**Correction: Journal of Neurology (2024) 271:5864–5874** 10.1007/s00415-024-12544-5

In the original version of this article, in Fig. 4 segmental heatmap is wrong. In particular, baseline STIR is not overlapped with T1 score (blue scale) that is the only one shown in the figure right now.

Figure 4 previously appeared as

**Figure:**
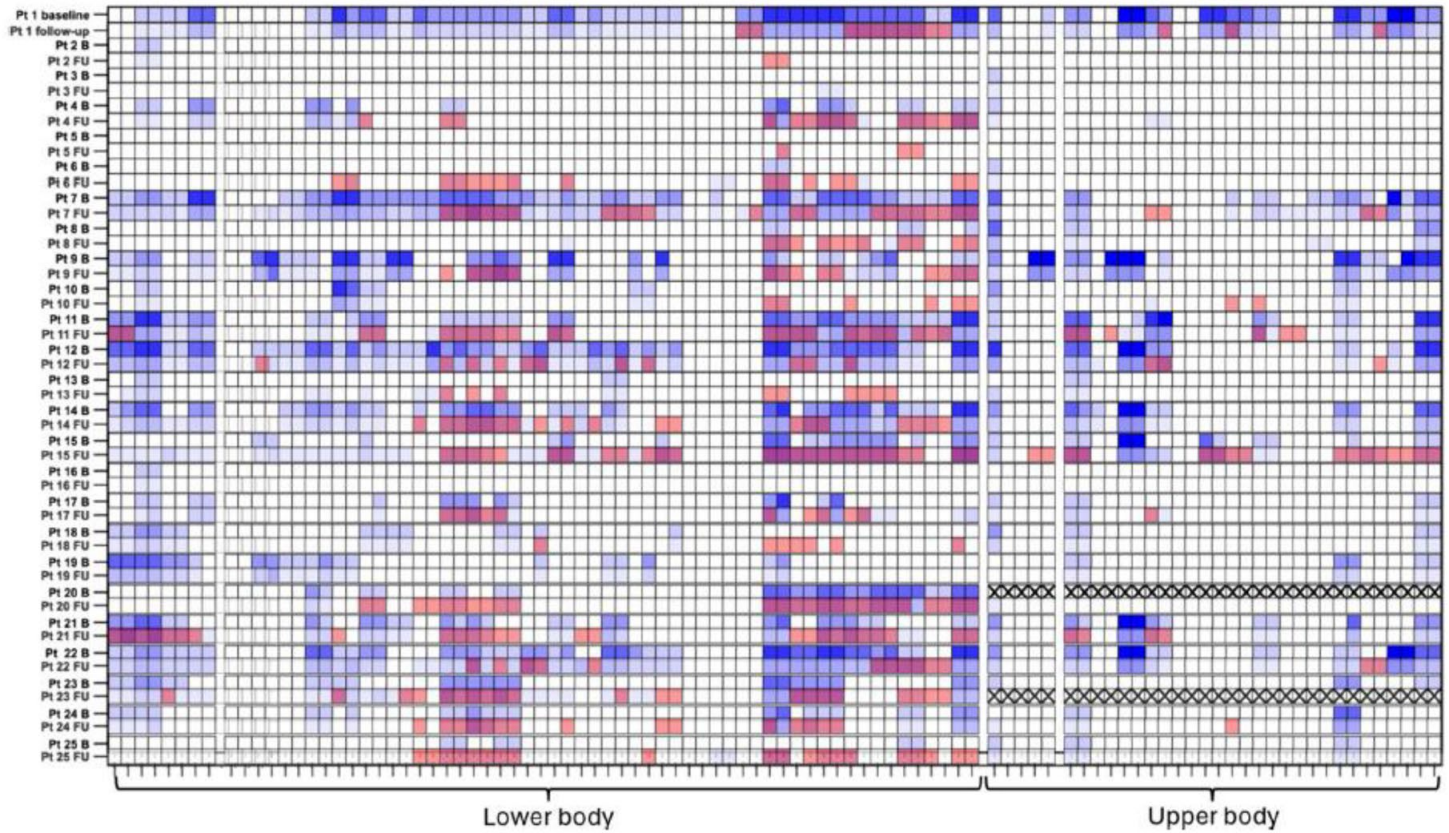
**Fig. 4** Segmental Heatmap. Overlapped STIR positivity (red or not) and T1-score (blue scale) of at BL and at FU for each patient and each muscle; note the good overlapping between T1 progression (darker blu) at FU and STIR positivity at BL and FU

But Fig. 4 should have appeared as shown below.

**Figure:**
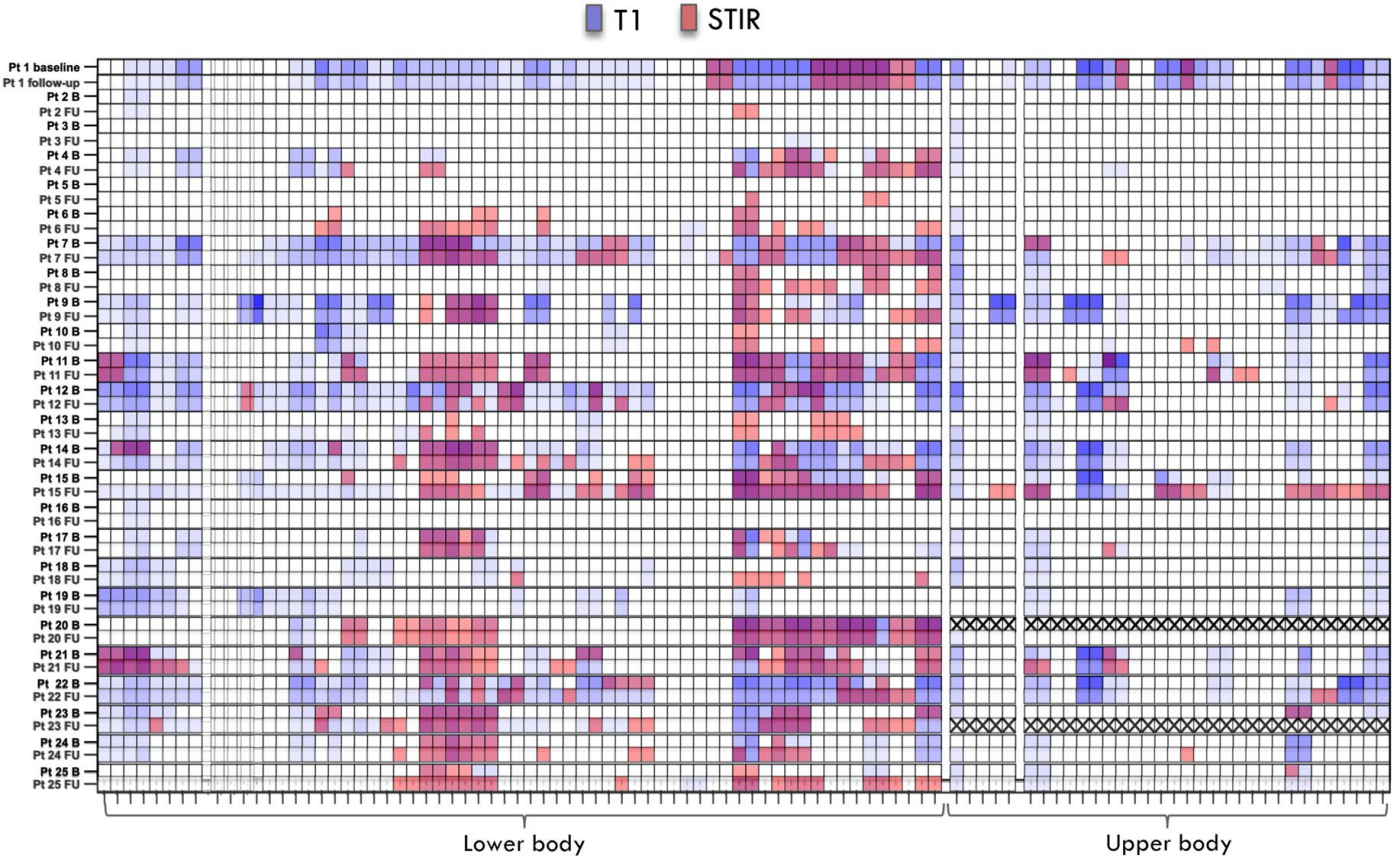
**Fig. 4** Segmental Heatmap. Overlapped STIR positivity (red or not) and T1-score (blue scale) of at BL and at FU for each patient and each muscle; note the good overlapping between T1 progression (darker blu) at FU and STIR positivity at BL and FU

